# Supplementary material for: SyNDI: synchronous network data integration framework
Source: BMC Bioinformatics. 2018 Nov 6;19:403. doi: 10.1186/s12859-018-2426-5 (PMC6219086; doi:10.1186/s12859-018-2426-5)
Supplement: Supplementary file 5 — Files of the example presented in Additional file 4. This additional file is a zip package that contains all files used in Additional file 4. Detailed descriptions of each of these files are presented in a README file included in this zip package. (GZ 30411 kb) [file 12859_2018_2426_MOESM5_ESM.gz › SyNDITools/Meme2Fimo/meme_4.9.0_4.tar.gz!/meme_4.9.0/doc/meme-chip.html]

MEME ChIP - motif discovery in ChIP-seq datasets


# The MEME Suite

## Motif-based sequence analysis tools

### Usage:

`meme-chip [options] [-db <motif database>]+ <sequences>`

### Description

MEME-ChIP performs several motif analysis steps on a set of DNA
sequences that you provide. It is especially appropriate for analyzing
the bound genomic regions identified in a transcription factor (TF)
ChIP-seq experiment.

MEME-ChIP can

1. discover novel DNA-binding motifs (with MEME and DREME),
2. determine which motifs are most centrally enriched (with CentriMo),
3. analyze them for similarity to known binding motifs (with Tomtom), and
4. automatically group the found motifs by similarity.

It is worth noting that MEME-ChIP is **not** a motif scanner, but
the motifs discovered by it can be used by FIMO or MAST to scan
for motif sites.

### Input

A set of sequences in FASTA format. Ideally the sequences should be all
the same length, between 100 and 500 base-pairs long and centrally
enriched for motifs. The immediate regions around individual ChIP-seq
"peaks" from a transcription factor (TF) ChIP-seq experiment are ideal.
The suggested 100 base-pair minimum size is based on the typical
resolution of ChIP-seq peaks but it is useful to have more of the
surrounding sequence to give CentriMo the power to tell if a motif is
centrally enriched. We recommend that you "repeat mask" your
sequences, replacing repeat regions to the "N" character.

### Output

MEME ChIP runs each program in its analysis in a different folder
in the output directory. A summary file (index.html) is created in
the output directory which lists the top motifs found and links to the
results for each program.

### Options

| Option | Parameter | Description | Default Behaviour |
| --- | --- | --- | --- |
| General Options | | | |
| --- | --- | --- | --- |
| -o | name | Create a folder called name and write output files in it. This option is not compatible with -oc as only one output folder is allowed. | The program behaves as if `-oc memechip_out` had been specified. |
| -oc | name | Create a folder called name but if it already exists allow overwriting the contents. This option is not compatible with -o as only one output folder is allowed. | The program behaves as if `-oc memechip_out` had been specified. |
| -index-name | name | Set the file name for the output summary to name. | The output summary is named index.html . |
| -db | file | Use file containing a database of DNA motifs in MEME format. This database will used by Tomtom and CentriMo. This option may be used multiple times to pass multiple databases. | When no databases are provided Tomtom can't suggest similar motifs and CentriMo is limited to the discovered motifs. |
| -bfile | file | Use file specifying background frequencies with programs that support a background (MEME, Tomtom and CentriMo). | A background file is calculated from the input sequences and passed to the programs which support it. |
| -nmeme | limit | The upper bound on the number of sequences that are passed to MEME. This is required because MEME takes too long to run for very large sequence sets. | The number of sequences passed to MEME will be limited to 600. |
| -ccut | size | The maximum length of a sequence to use before it is trimmed to a central region of this size. A value of 0 indicates that sequences should not be cut down. | A maximum size of 100 is used. |
| -time | minutes | The maximum time that MEME-ChIP has to run and create output. | There is no time limit |
| -desc | description | A description of the MEME ChIP run which is displayed in the summary file. | No description is displayed in the summary file. |
| -fdesc | file | A file containing a description of the MEME ChIP run which will be included in the summary file. The summary file will try to preserve some of the formatting by presenting blocks of text separated by multiple new lines as different paragraphs and replacing single new line characters with line breaks. Only the first 500 characters are used. | No description is displayed in the summary file. |
| -norc |  | Find motifs in given strand only. | Find motifs in both strands. |
| -noecho |  | Don't echo the commands run. | Echo the commands run to standard output. |
| -help |  | Display a usage message |  |
| MEME Specific Options | | | |
| --- | --- | --- | --- |
| -meme-mod | oops\|zoops\|anr | The number of motif sites that MEME will find per sequence. **oops** - One Occurrence Per Sequence,  **zoops** - Zero or One Occurrence Per Sequence,  **anr** - Any Number of Repetitions See -mod in the MEME command-line documentation. | MEME defaults to using zoops mode. |
| -meme-minw | width | The minimum motif width that MEME should find. | A minimum width of 6 is used unless the maximum width has been set to be less than 6 in which case the maximum width is used. |
| -meme-maxw | width | The maximum motif width that MEME should find. | A maximum width of 30 is used unless the minimum width has been set to be larger than 30 in which case the minimum width is used. || -meme-nmotifs | num | The number of motifs that MEME should search for. | MEME will find 3 motifs. |
| -meme-minsites | sites | The minimum number of sites that MEME needs to find for a motif. | MEME doesn't require any minimum number of sites for a motif. |
| -meme-maxsites | sites | The maximum number of sites that MEME will find for a motif. | MEME doesn't limit the number of sites it will find for a motif. |
| -meme-p | np | Use parallel version of meme with np processors |  |
| -meme-pal |  | Restrict MEME to searching for palindromes only. | MEME searches for any motif not just palindromes. |

| DREME Specific Options | | | |
| --- | --- | --- | --- |
| -dreme-e | *E*-value | Stop searching for more motifs if the next best motif found has a worse *E*-value | An *E*-value threshold of 0.05 is used. |
| -dreme-m | count | Stop searching for more motifs if count motifs have been found. | There is no limit on the number of motifs. |
| CentriMo Specific Options | | | |
| --- | --- | --- | --- |
| -centrimo-local |  | CentriMo perform *local* motif enrichment analysis, computing enrichment in every possible sequence region. | CentriMo will perform *central* motif enrichment analysis, computing enrichment in centered regions only. || -centrimo-score | score | Set the minimum accepted score for a match. | A minimum score of 5 is used. |
| -centrimo-maxreg | region | Set the size of the maximum region size tested. | CentriMo will test all valid region sizes. |
| -centrimo-ethresh | *E*-value | Set the *E*-value threshold for reporting enriched central regions. | An *E*-value threshold of 10 will be used. |
| -centrimo-noseq |  | Do not store sequence IDs in the output of CentriMo. | CentriMo stores a list of the sequence IDs with matches in the best region for each motif. |
